# Increase in healthcare utilization and Medicare payment with progression of preclinical Alzheimer’s disease

**DOI:** 10.1016/j.tjpad.2026.100547

**Published:** 2026-04-01

**Authors:** Julie Beyrer, Zachary Sheff, Nalin Payakachat, Julie M. Chandler, Yun-Fei Chen, Joanna Kubisiak, Angelina Lee, Karen C. Holdridge, Roy Yaari, Paul Aisen, Michael S. Rafii, Reisa A. Sperling

**Affiliations:** a Eli Lilly and Company, Indianapolis, IN, United States; b Westat, Rockville, MD, United States; c University of Southern California, San Diego, CA, United States; d Brigham and Women’s Hospital, Massachusetts General Hospital, Harvard Medical School, Boston, MA, United States; e A4 and LEARN Study Teams - Alzheimer Clinical Trials Consortium

**Keywords:** A4, LEARN, Alzheimer’s disease, Health resource utilization, AD disease progression, Medicare, Cognitively unimpaired (preclinical) AD

## Abstract

**Background:**

Alzheimer’s disease (AD) begins with the preclinical stage (Stages 1 and 2) where individuals are cognitively unimpaired but have AD pathology. Healthcare utilization and medical cost of cognitively unimpaired (preclinical) AD have not been previously evaluated.

**Objectives:**

To describe healthcare resource utilization (HRU) and Medicare payments among cognitively unimpaired individuals with and without elevated amyloid and to evaluate the association of AD progression with HRU and Medicare payments.

**Design:**

Retrospective cohort analysis of the randomized controlled trial (Anti-Amyloid Treatment in Asymptomatic AD [A4]) and companion observational study (Longitudinal Evaluation of Amyloid Risk and Neurodegeneration [LEARN]) linked with Medicare.

**Setting:**

Clinical trials sites in the United States

**Participants:**

246 cparticipants with cognitively unimpaired AD in A4 and 121 amyloid-negative participants in LEARN Medicare cohorts.

**Measurements:**

Measures from Medicare claims included medical conditions (diagnosis codes), HRU (inpatient, emergency room, outpatient, professional, skilled nursing facility, home health), and Medicare payments. AD progression (or cognitive or functional decline) was measured using Clinical Dementia Rating Scale-Global Score (CDR-GS) in A4/LEARN and diagnosis codes for cognitive impairment, AD, and JEN Frailty Index (JFI) of ≥6 (high frailty) in Medicare data.

**Results:**

HRU and payments were overall similar between A4 and LEARN Medicare. Claims indicators suggesting AD progression in A4 Medicare were associated with higher inpatient, outpatient, emergency room, and home health utilization (any utilization) and increased number of inpatient stays. Payments were significantly greater in A4 Medicare with AD progression vs without progression: 45% payment increase for cognitive impairment (p=0.035) with a mean incremental cost of $140 per person per month (PPPM) (95% confidence interval [CI] $125-$155), 66% payment increase for AD (p=0.011) with a mean incremental cost of $207 PPPM (95% CI $188-$226), and 103% payment increase for high frailty (p<0.001) with a mean incremental cost of $303 PPPM (95% CI $283-$323).

**Conclusions:**

Overall, individuals with cognitively unimpaired AD in A4 Medicare did not have increased utilization and payments vs LEARN. HRU and payments were significantly greater in A4 Medicare participants with AD progression indicators in claims vs those without progression. These results highlight the need for additional research on both health and economic impacts of progression in a real-world (routine care) cohort of individuals with cognitively unimpaired AD and the potential cost savings associated with effective therapy that delays AD progression in cognitively unimpaired AD.

**Clinical trial registration information:**

NCT02008357, NCT02488720

## 1 Introduction

Alzheimer’s disease (AD) encompasses a biological and clinical continuum that includes the preclinical stage (unimpaired cognition with AD pathology; also known as Stages 1 and 2) [1,2], the mild cognitive impairment (MCI) stage (often called prodromal) with objective impairment in at least one cognitive domain, and the dementia stage with multiple cognitive impairments [3]. Although AD is historically described by clinical syndrome presentations, more recently, a biologically based diagnosis has been advanced to assist in staging and early identification of individuals with AD [1].

Cognitively unimpaired (preclinical) AD presents with an accumulating brain pathology (i.e., amyloid beta [Aβ] plaques and hyperphosphorylated tau into paired helical filament neurofibrillary tangles) [4]. In addition, individuals with cognitively unimpaired AD have evidence of abnormal biomarkers (amyloid positron emission tomography [PET], cerebrospinal fluid [CSF] biomarkers, and plasma biomarkers such as phosphorylated tau 217 [P-tau217]) associated with AD pathology [1,5]. In cognitively unimpaired AD, early signs of neurodegeneration (e.g., elevated CSF tau, mild cortical thinning) are often present, but irreversible neuronal loss is not yet widespread [1,5]. Individuals with cognitively unimpaired AD have no objective cognitive decline, but subtle changes can be detected with sensitive neuropsychological tests [1,2,5]. Subjective cognitive decline may appear in this stage.

Cognitively unimpaired AD may precede AD with MCI by several years or more than a decade [6] and represents an important stage for potential early therapeutic intervention aimed at slowing the pathophysiological process and delaying the appearance of readily appreciated clinical manifestations of AD [1]. Based on results of previous clinical trials, it is hypothesized that treatment in the early stages of AD with amyloid-targeting therapies could more effectively delay progression [7].

The global prevalence of cognitively unimpaired AD is substantial with an estimated prevalence based on Aβ PET imaging positivity of 315 million (17% prevalence among individuals aged 50 years and above) [8]. In the United States (U.S.), the prevalence of cognitively unimpaired AD (Aβ positive) was estimated to be 6.7 million persons or 16.3% among 55 to 64 year olds and 9.9 million persons or 22.1% among 65 to 79 year olds [[9], [10], [11], [12], [13], [14]]. A previous analysis of the Anti-Amyloid Treatment in Asymptomatic Alzheimer’s Disease (A4) trial showed that participants in the highest tertile of plasma P-tau217 at baseline had a greater risk of cognitive and functional decline than those in lower tertiles [11]. Using these data, an estimated 2.2 million people aged 55 to 64 years (5.4% prevalence) and 3.3 million people aged 65 to 79 years (7.4% prevalence) would be at a greater risk of cognitive decline and progression to symptomatic AD in the U.S [[9], [10], [11], [12], [13], [14], [15]].

Although the economic burden of cognitively unimpaired AD is relatively unknown, symptomatic stages of AD impose a significant economic burden on the healthcare system. Payments for services for individuals aged ≥65 years with dementia show total payments for healthcare, long-term care, and hospice services estimated to exceed $360 billion [16]. Accordingly, AD is a major cause of disability globally with a mounting social and financial impact not only for individuals, families, and care partners but also for health and social care systems.

While there is some evidence on healthcare resource utilization (HRU) and cost in early symptomatic AD [[16], [17], [18], [19], [20], [21]], knowledge gaps persist for understanding the economic impact of cognitively unimpaired AD. A hypothetical model to assess potential economic impact of treating cognitively unimpaired AD showed that treatment could be economically viable from a payer and societal perspective [22]. However, actual HRU andcosts data in cognitively unimpaired AD are lacking. Given the higher cost associated with disease progression and growing level of impairment in symptomatic stages of AD, it is also important to understand the economic impact in the cognitively unimpaired (preclinical) stage of AD.

The current paper examines healthcare utilization and direct medical costs of cognitively unimpaired AD using Medicare claims from participants in the Anti-Amyloid Treatment in Asymptomatic AD (A4) trial and the Longitudinal Evaluation of Amyloid Risk and Neurodegeneration study (LEARN) companion observational study. Specifically, this paper describes findings from the retrospective, observational study of the A4 and LEARN Medicare addendum, focusing on changes that occurred during the on-study period, including a) comparison of clinical characteristics, HRU, and payments among cognitively unimpaired individuals with elevated amyloid (i.e., cognitively unimpaired AD in A4 Medicare) and individuals without elevated amyloid (LEARN Medicare) and b) evaluation of the association of AD progression with HRU and Medicare payments in A4 Medicare.

## 2 Methods

### 2.1 Study design

The A4 trial, a randomized, double-blind, placebo-controlled, Phase 3 trial, was designed to test the efficacy of solanezumab, a monoclonal antibody, at slowing cognitive decline in individuals living with cognitively unimpaired AD. LEARN, a companion observational study, enrolled individuals who met all the A4 inclusion criteria but who were ineligible for the A4 trial due to the absence of elevated amyloid on their PET scans [11]. LEARN characterized the longitudinal changes in the cognitive, clinical, and biomarker measures used in the A4 trial in a population without elevated amyloid, serving as a control cohort for comparison with the A4 trial population [23,24]. Both A4 and LEARN study populations were aged 65 to 85 years. To study economic outcomes in relation to AD progression and amyloid status, some of the A4 and LEARN participants in the U.S. consented to have their clinical study data linked with Medicare claims data.

This retrospective observational study followed 246 A4 participants who were cognitively unimpaired (based on Clinical Dementia Rating Scale-Global Score [CDR-GS] of 0 and education-adjusted Mini Mental State Examination [MMSE] score of 25 to 30) and met the elevated brain amyloid eligibility criteria established via ^18^F-florbetapir PET imaging and 121 participants in the companion LEARN study without elevated brain amyloid (see Section 2.2 for addendum details). A4 or LEARN study data were linked with Medicare claims data for up to 5 years prior to A4 or LEARN study entry (pre-study period), during the on-study period, and up to 10 years after the study for A4 and up to 7 years after for LEARN, hereinafter referred to as the A4 and LEARN Medicare addendum.
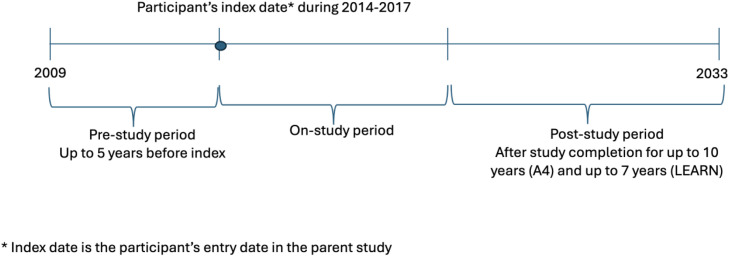


Data in our analyses include the pre-study period and on-study period. Two analytic cohorts are described for this addendum study: Utilization Cohort and Payment Cohort. The inclusion criteria for each study cohort and the types of data available are summarized in **Suppl Table 1**.

### 2.2 Addendum study population

The addendum population included adults who participated in the A4 and LEARN studies [11,23,24] if they met all of the following criteria: were randomized participants in A4 and received at least 1 dose of study drug or were participants in LEARN who had at least 1 study visit; were enrolled in Medicare at the time of entry into A4 or LEARN; and were willing to provide personal identifying information to the site study team to enable the linkage of their study data with Medicare data.

The A4 and LEARN studies were conducted in accordance with the International Conference on Harmonisation guidelines and ethical principles of the Declaration of Helsinki. The Medicare addenda of the A4 and LEARN studies were approved by the institutional review board or independent ethics committee at each participating center. All participants, caregivers, or the participant’s legal representative provided written informed consent.

### 2.3 Outcome measures

*Medical conditions:* International Classification of Disease, 10^th^ revision, (ICD-10) diagnosis codes relevant to early AD symptoms were reported from the Medicare fee for service (FFS) and Medicare Advantage (MA) data to describe participants’ medical conditions.

*HRU:* The FFS claims and MA encounter records were categorized to create HRU measures for inpatient, emergency room, skilled nursing facility (SNF), and home health utilization, as well as for the different types of professional services. Healthcare Common Procedure Coding System Level I Codes (also known as Current Procedural Terminology [CPT®] codes) were used to categorize medical encounters across all care settings. Two HRU measures are reported: 1) the proportion of the cohort with utilization in each category and 2) the average per participant per month (PPPM) measure of utilization units (i.e., episodes, days, visits, and encounters) in the period (e.g., the entire on-study period or the specified year of a period) [25]. For each participant, the calculation of the PPPM measure is summarized in the equation below.

$$PPPM\;utilization\;measure_{p}=\;\frac{\sum Utilization\;Units_{m}}{Medicare\;Part\;A\;and\;B\;Covered\;Months_{p}}$$

where p=observation period, m=observation month within the period; units include episodes, visits, and days

To facilitate presentation and interpretation, the profiles also include the annualized rate of events per 100 participants, which is calculated by multiplying the PPPM estimate by 1200. Because a participant may contribute varying numbers of months to each observation period, this standardization allows for interpretations (e.g., over the entire pre-study period, on average, there were approximately [insert number here] number of hospital episodes per year for every 100 participants).

*Medicare payments*: Medicare Part A and B FFS claims were adjusted for inflation to 2021 U.S. dollars based on Centers for Medicare & Medicaid Services (CMS) National Health Expenditure Accounts annual personal health care estimates for Medicare [26]. Average PPPM payment measures were summarized over the study periods.

*Disease progression*: In the A4 and LEARN studies, AD progression was defined as two consecutive, or one final, CDR-GS ≥0.5. Three additional indicators suggesting AD progression were extracted from Medicare data, defined using ICD-10 diagnosis codes for cognitive impairment diagnosis, AD diagnosis, and high frailty based on the JEN Frailty Index (JFI).

The list of ICD-10 codes used to define cognitive impairment and AD diagnosis in Medicare claims (AD progression indicators) is provided in **Suppl Table 2**.

The JFI is a claims-based measure of frailty that includes comorbid medical conditions related to cognition and function [27]. The JFI groups ICD-9/10 diagnostic codes into 13 categories, consisting of minor ambulatory limitations, severe ambulatory limitations, cognitive developmental disability, chronic mental illness, dementia, sensory disorders, self-care impairment, syncope, cancer, chronic medical disease, pneumonia, renal disorders, and other systemic disorders [28]. The JFI score is calculated by summing across the 13 categories and categorized into low frailty capturing scores 0 to 3, medium frailty capturing scores 4 to 5, and high frailty capturing scores 6 to 13. JFI scores have been correlated with functional status (i.e., activities of daily living), high-payment utilization, nursing facility entry, and mortality [[28], [29], [30], [31]]. In the GERAS-US study [32], participants with mild dementia had higher JFI scores than those with MCI. A JFI score ≥6 was used to identify high frailty in this study, which has been previously correlated with having ≥2 activities-of-daily-living dependencies [28]. The JFI score at the end of each observation period (pre-study and on-study) is based on diagnoses observed during the current month and previous 11 months.

### 2.4 Statistical analysis

Demographic characteristics of A4 and LEARN participants for the Medicare Addendum study were described and compared using Chi-square tests for categorical variables and from *t*-tests for continuous variables. Data were suppressed when <11 participants were identified or could be derived in ≥1 category per the CMS cell size suppression policy. Statistically significant differences (p<0.05) were noted.

The frequency of medical conditions was described and compared between A4 and LEARN Medicare study cohorts using Chi-square tests. Unadjusted Kaplan-Meier (KM) survival curves for time to disease progression were created for the on-study period. For CDR-GS, the time to first score increase was confirmed by a second increase in score, unless it was the last CDR-GS during the on-study period, in which case one increase was sufficient for identifying progression.

A multivariable Cox proportional hazards model was fit and adjusted for age and sex.

Differences in HRU rates were compared between A4 and LEARN Medicare using Wilcoxon rank sum tests, and the estimates of proportions with utilizations were assessed with Chi-square tests. To evaluate descriptive differences in PPPM payments between A4 and LEARN Medicare, given the skewness of the payment data, bootstrapping with 10,000 iterations was performed to establish 95% confidence intervals (CI) for the payment estimates in each study cohort, as well as for the difference in mean payment estimates between the study cohorts.

After identifying the upper quartile for PPPM total Medicare payments for A4 Medicare participants during the on-study period, the relationship between having higher payments and the four varying AD progression indicators was analyzed. Each of the four varying AD progression indicators was analyzed separately using logistic regression for the binary outcome (progression/no progression) with adjustments for age and sex. For the JFI, a score of ≥6 (high frailty) during the participant’s final on-study month and previous 11 months was used to define progression. After observing the association between AD progression and the probability of high Medicare cost through logistic regression, log-normal linear regression models examined the relationship between log-transformed PPPM total Medicare payments and each of the four varying AD progression indicators, with adjustments for age and sex. The log of the PPPM total Medicare spending was used as the outcome variable to improve the normality and homoscedasticity of the residuals, and Winsorization to the 99th percentile was used to reduce the effect of extreme outliers [33]. Mean predicted PPPM payments for non-progressors were subtracted from those for progressors to derive the incremental PPPM cost differences.

All analyses for the Medicare Addendum were conducted within the CMS Virtual Research Data Center using SAS Version 9.4.

## 3 Results

### 3.1 Demographics

Baseline (end of pre-study period) demographic information is summarized in **Suppl Table 3**. The mean age of A4 Medicare was significantly higher than that of LEARN Medicare (72.3 vs 70.8 years, respectively; p=0.004) in the year of study entry. A4 and LEARN Medicare differed in their distribution by region (p<0.001) and county classification (p=0.011), wherein LEARN participants were more highly concentrated in the Northeast and large central metropolitan counties (49% and 37%, respectively) compared with A4 (30% and 27%, respectively). A4 and LEARN Medicare did not differ in their distribution by race; most participants were white. At entry into their respective parent studies, A4 Medicare had a lower mean number of years of education (16.7 vs 17.4 years, respectively; p=0.009) than LEARN Medicare. A4 Medicare had a significantly greater proportion of participants reporting a family history of dementia–parent or sibling than LEARN Medicare (76% and 66%, respectively; p=0.045) and a significantly greater proportion of *APOE ε4* carriers (62% and 12%, respectively; p<0.001).

### 3.2 Baseline (pre-study period)

#### 3.2.1 Clinical characteristics

Baseline comorbidities and JFI were comparable between participants with elevated amyloid (A4 Medicare) and without elevated amyloid (LEARN Medicare) (Table 1). Disorders of lipoprotein metabolism and other lipidemias (A4: 72%, LEARN: 73%; OR 0.98 [95% CI 0.59-1.63]) and hypertension (A4: 55%, LEARN: 47%: OR 1.34 [95% CI 0.85-2.12]) were the most prevalent conditions in both cohorts. Hypothyroidism (A4: 26%, LEARN: 13%; OR 2.46 [95% CI 1.31-4.64]) was the only medical condition that was statistically different between the two cohorts at baseline.

**Table 1:** Comparison of clinical characteristics and outcomes for A4 & LEARN Medicare cohorts at baseline (end of pre-study) and end of on-study periods.

	ICD Code	Baseline conditions/risk factors			End of on-study conditions		
Condition / Risk Factor List		A4	LEARN	OR (LCL - UCL)	A4	LEARN	OR (LCL - UCL)
Major depressive disorder	F32	15%	-	-	26%	22%	1.26 (0.75 - 2.12)
Anxiety	F41	13%	14%	0.96 (0.50 - 1.88)	26%	26%	0.96 (0.58 - 1.58)
Reaction to severe stress and adjustment disorders	F43	8%	-	-	15%	19%	0.74 (0.41 - 1.34)
Other symptoms and signs involving cognitive functions and awareness	R41	13%	-	-	31%	18%	2.02 (1.17 - 3.49)*
Malaise and fatigue	R53	12%	10%	1.22 (0.58 - 2.55)	49%	50%	0.98 (0.63 - 1.53)
Abnormal weight loss	R63.4	-	-	-	18%	15%	1.21 (0.66 - 2.21)
Overweight and obesity	E66	18%	15%	1.17 (0.63 - 2.17)	32%	29%	1.15 (0.71 - 1.86)
Disorders of lipoprotein metabolism and other lipidemias	E78	72%	73%	0.98 (0.59 - 1.63)	88%	86%	1.14 (0.59 - 2.20)
Other hypothyroidism	E03	26%	13%	2.46 (1.31 - 4.64)*	30%	21%	1.60 (0.95 - 2.69)
Sleep disorders	G47	25%	19%	1.42 (0.81 - 2.49)	42%	33%	1.47 (0.93 - 2.34)
Other cerebrovascular diseases	I67	-	-	-	9%	-	-
Other disorders of urinary system	N39	23%	21%	1.12 (0.65 - 1.96)	38%	26%	1.80 (1.10 - 2.93)*
Fracture of rib(s), sternum and thoracic spine	S22	-	-	-	5%	-	-
Fracture of forearm	S52	-	-	-	5%	-	-
Fracture unspecified	T14	-	-	-	11%	9%	1.18 (0.56 - 2.48)
Slipping, tripping, stumbling and falls	W00-W19	8%	-	-	18%	24%	0.68 (0.40 -1.17)
Headache	R51	-	-	-	16%	21%	0.72 (0.41 -1.26)
Migraine	G43	5%	-		11%	-	-
Essential (primary) hypertension	I10	55%	47%	1.34 (0.85 - 2.12)	73%	59%	1.89 (1.19 - 3.02)*
Elevated blood glucose level	R73	20%	22%	0.89 (0.51 -1.55)	48%	37%	1.58 (1.00 -2.49)*
Type 2 diabetes mellitus	E11	18%	18%	0.96 (0.53 - 1.74)	20%	15%	1.39 (0.77 - 2.52)
Ischemic heart disease	I25	17%	15%	1.22 (0.65 - 2.29)	29%	27%	1.06 (0.65 - 1.74)
Atrial fibrillation and flutter	I48	8%	-	-	16%	14%	1.24 (0.66 - 2.32)
Cerebral infarction	I63	-	-	-	6%	-	-
Visual disturbances and blindness	H53-H54	10%	-	-	21%	27%	0.72 (0.43 - 1.21)
Conductive and sensorineural hearing loss	H90	19%	18%	1.08 (0.60 - 1.94)	39%	38%	1.05 (0.66 - 1.65)
Other and unspecified hearing loss	H91	10%	-	-	29%	25%	1.21 (0.73 - 2.01)
Alzheimer's disease	G30	-	-	-	24%	-	-
Mild cognitive impairment	G31	-	-	-	21%	-	-
**JFI Measure**		**Baseline JFI**			**End of on-study JFI**		
		**A4**	**LEARN**	**p-value**	**A4**	**LEARN**	**p-value**
JFI Score (mean, SE)		3.00 (0.12)	2.81 (0.15)	0.339	4.58 (0.13)	3.90 (0.19)	0.003*
JFI Score-Low (JFI 0 to 3)		64% (3%)	66% (5%)	0.287	28% (3%)	44% (5%)	0.009*
JFI Score-Medium (JFI 4 to 5)		25% (3%)	-	-	42% (3%)	33% (4%)	-
JFI Score-High (JFI 6+)		11% (2%)	-	-	30% (3%)	22% (4%)	-
**Cognitive Assessment**		**Baseline cognitive**			**End of on-study cognitive**		
		**A4**	**LEARN**	**p-value**	**A4**	**LEARN**	**p-value**
CDR-Global (mean, SE)		0.00 (0.00)	0.00 (0.00)	-	0.22 (0.02)	0.06 (0.02)	<0.001*
CDR-SOB (mean, SE)		0.03 (0.01)	0.03 (0.01)	0.968	0.79 (0.10)	0.13 (0.03)	<0.001*
CFI (mean, SE)		1.99 (0.11)	1.76 (0.17)	0.254	2.76 (0.17)	2.08 (0.18)	0.006*
Mini Mental State Exam (mean, SE)		28.81 (0.08)	29.09 (0.10)	0.031*	27.99 (0.24)	29.18 (0.10)	<0.001*
Wechsler Memory Scale LMDR Score (mean, SE)		13.21 (0.23)	14.24 (0.31)	0.009*	13.04 (0.36)	15.55 (0.34)	<0.001*
CFI combined score (mean, SE)		3.24 (0.19)	2.83 (0.26)	0.209	5.12 (0.33)	3.14 (0.26)	<0.001*

ADL = Activities of Daily Living; CDR = Clinical Dementia Rating Scale; SOB = sums of boxes; CFI = Cognitive Function Index; CI = confidence interval; ICD = International Classification of Diseases; JFI = JEN Frailty Index; LCL = lower confidence level; LMDR=Logical Memory Delayed Recall, OR = odds ratio; PACC = Preclinical Alzheimer's Cognitive Composite; SE = standard error; UCL = upper confidence level

Notes: Data reported for the “Utilization” cohort. The JFI score at the end of each observation period (pre-study or on-study) is based on diagnoses observed during the final month in that time period and the previous 11 months. The top panel provides prevalence of conditions based on ICD-10 codes at the end of the pre-study period (Baseline) and end of on-study period for each cohort. ORs with 95% CIs are provided using A4 Medicare as the reference group. ORs that are statistically significant (p-value <0.05) are indicated with an asterisk. The middle panel compares JFI scores and categories between cohorts at baseline and end of on-study period. The bottom panel provides cognitive assessment scores for each cohort at baseline and end of study period. Cells containing less than 11 individuals are suppressed per Medicare policy and denoted with a dash.

⁎ Bold-faced numbers represent significant findings.

A4 and LEARN Medicare had similar mean JFI scores at baseline (A4: 3.00, LEARN: 2.81; p=0.339), and most participants were categorized with low frailty (A4: 64%, LEARN: 66%; p=0.287) (Table 1).

#### 3.2.2 Cognitive assessments

Per study protocol inclusion criteria, A4 and LEARN participants did not have cognitive impairment upon enrollment based on cognitive assessment scores [11,23]. However, even within this normal range, A4 Medicare participants had a lower mean baseline MMSE score compared with LEARN Medicare participants (28.81 vs 29.09, respectively; p=0.031) (Table 1).

#### 3.2.3 HRU

Baseline HRU did not significantly differ between A4 and LEARN Medicare in terms of the proportions of participants utilizing care in inpatient, emergency room, and outpatient settings (Table 2). However, A4 Medicare had a significantly higher proportion of participants with an anesthesia encounter (49% vs 35%, respectively; p=0.010) and a significantly higher annualized rate per 100 participants of anesthesia encounters (34 vs 21, respectively; p=0.008) and outpatient ER visits (21 vs 10, respectively; p=0.028) compared with LEARN Medicare (Table 2). In addition, A4 Medicare had a significantly higher rate of surgery encounters (322 vs 260, respectively; p=0.040) and a significantly higher rate of encounters for evaluation and management services (728 vs 603, respectively; p=0.039) compared with LEARN Medicare (Table 2).

**Table 2:** Comparison of health-related utilization for A4 & LEARN Medicare cohorts in the pre-study and on-study periods.

	Baseline (pre-study period)*			On-study period		
Percent with any utilization	A4	LEARN	p-value	A4	LEARN	p-value
**Type of utilization**	% (SE)	% (SE)		% (SE)	% (SE)	
Inpatient Episodes	20% (3%)	12% (3%)	0.056	32% (3%)	29% (4%)	0.582
Inpatient Days	20% (3%)	12% (3%)	0.056	32% (3%)	29% (4%)	0.582
Home Health Days	5% (1%)	-	-	19% (3%)	18% (4%)	0.827
Inpatient ER Visits	8 % (2%)	-	-	17% (2%)	15% (3%)	0.661
Outpatient ER Visits	35% (3%)	25% (4%)	0.071	56% (3%)	50% (5%)	0.232
Non-ER Outpatient Visits	83% (3%)	85% (3%)	0.690	95% (1%)	91%^	-
Professional encounters						
Anesthesia	49% (3%)	35% (5%)	**0.010**†	87% (2%)	80% (4%)	0.080
Surgery	91% (2%)	87% (3%)	0.263	95%^	91%^	-
Radiology Procedures	87% (2%)	87% (3%)	0.901	95%^	91%^	-
Pathology and Laboratory Procedures	88% (2%)	87% (3%)	0.919	95%^	91%^	-
Medicine Services and Procedures	91% (2%)	90%^	-	100%^	100%^	-
Evaluation and Management Services	95%^	90%^	-	100%^	100%^	-
**Annualized rate of utilization per 100 participants**						
**Type of utilization**	Rate (SE)	Rate (SE)		Rate (SE)	Rate (SE)	
Inpatient Episodes	7 (1)	4 (1)	0.060	13 (5)	14 (5)	0.693
Inpatient Days	29 (6)	16 (5)	0.072	60 (16)	78 (32)	0.812
Home Health Days	37 (12)	-	-	172 (41)	103 (25)	0.848
Inpatient ER Visits	3 (1)	-	-	7 (3)	4 (1)	0.683
Outpatient ER Visits	21 (3)	10 (2)	**0.028**†	21 (2)	21 (5)	0.161
Non-ER Outpatient Visits	335 (29)	405 (56)	0.136	424 (28)	545 (42)	**0.002**†
Professional encounters						
Anesthesia	34 (3)	21 (3)	**0.008**†	60 (4)	56 (5)	0.518
Surgery	322 (18)	260 (20)	**0.040**†	363 (15)	330 (21)	0.197
Radiology Procedures	215 (13)	192 (15)	0.460	242 (16)	273 (30)	0.445
Pathology and Laboratory Procedures	241 (15)	197 (16)	0.126	277 (17)	198 (15)	**0.002**†
Medicine Services and Procedures	531 (40)	632 (121)	0.542	685 (36)	793 (106)	0.901
Evaluation and Management Services	728 (33)	603 (36)	**0.039**†	867 (37)	812 (51)	0.268

ER = Emergency room; SE = standard error

Notes: Utilization data obtained from the Medicare claims data “Utilization” cohort. HRU was collected over the entire observation period (pre-study or on-study). The top panel shows the proportion of participants in each cohort with any utilization for a given utilization category. The bottom panel show the annualized rate of utilization per 100 participants (i.e., the number of episodes/visits/procedures that would be expected in a year from a group of 100 participants). Cells containing less than 11 individuals are suppressed per Medicare policy and denoted with a dash. Cells with suppressed SEs are indicated with a ^.

⁎ Measured across entire pre-study period.

† Bold-faced numbers represent significant findings.

#### 3.2.4 Medicare payment

Baseline total payments were comparable between A4 and LEARN Medicare (A4 mean PPPM=$449; LEARN mean PPPM=$394; 95% CI difference in mean PPPM -$56 - $161) (Table 3). However, A4 Medicare had a statistically significantly higher payment for emergency room (A4 mean PPPM=$6; LEARN mean PPPM=$3, 95% CI difference in mean PPPM $1-$5) and for professional services related to anesthesia (A4 mean PPPM=$7; LEARN mean PPPM=$5, 95% CI difference in mean PPPM $0-$4).

**Table 3:** Comparison of payments for A4 & LEARN Medicare cohorts in the pre-study and on-study periods.

	Baseline (pre-study period)				On-study period			
	A4	LEARN	Difference	Significance	A4	LEARN	Difference	Significance
Payment Categories	PPPM Payment Mean (SE)	PPPM Payment Mean (SE)	PPPM Payment A4 - LEARN (95% CI)	p-value	PPPM Payment Mean (SE)	PPPM Payment Mean (SE)	PPPM Payment A4 - LEARN (95% CI)	p-value
Inpatient	$91 ($17)	$56 ($20)	$35 (-$17 - $85)	0.175	$286 ($171)	$335 ($202)	-$49 (-$618 - $486)	0.873
ER-Total	$6 ($1)	$3 ($1)	$3 ($1 - $5)	0.019*	$12 ($5)	$8 ($2)	$4 (-$5 - $16)	0.507
*ER-Inpatient*	$1 ($0)	$1 ($0)	$1 (-$1 - $2)	0.319	$8 ($5)	$5 ($2)	$3 (-$5 - $15)	0.544
*ER-Outpatient*	$4 ($1)	$2 ($1)	$2 ($0 - $4)	0.027*	$4 ($1)	$4 ($1)	$1 (-$3 - $3)	0.733
Outpatient	$105 ($13)	$113 ($14)	-$8 (-$46 - $29)	0.672	$169 ($30)	$144 ($16)	$25 (-$35 - $99)	0.470
DME	$5 ($1)	$8 ($4)	-$3 (-$13 - $3)	0.404	$7 ($2)	$9 ($5)	-$2 (-$14 - $6)	0.713
Home Health	$5 ($2)	$6 ($3)	-$1 (-$8 - $6)	0.893	$15 ($4)	$10 ($3)	$6 (-$3 - $15)	0.225
Hospice	$0 ($0)	$0 ($0)	$0 ($0 - $0)	-	$3 ($3)	$0 ($0)	$3 ($0 - $8)	0.193
SNF	$1 ($1)	$0 ($0)	$1 ($0 - $4)	0.143	$9 ($5)	$14 ($7)	-$5 (-$21 - $9)	0.504
Other	$0 ($0)	$0 ($0)	$0 ($0 - $0)	-	$0 ($0)	$0 ($0)	$0 ($0 - $0)	
Professional Services	$235 ($18)	$208 ($21)	$28 (-$29 - $80)	0.325	$307 ($48)	$281 ($38)	$26 (-$87 - $156)	0.682
*Anesthesia*	$7 ($1)	$5 ($1)	$2 ($0 - $4)	0.032*	$8 ($1)	$11 ($3)	-$3 (-$9 - $2)	0.387
*Evaluation and Management Services*	$54 ($3)	$45 ($4)	$8 (-$3 - $19)	0.139	$65 ($5)	$75 ($22)	-$9 (-$60 - $23)	0.671
*Medicine Services and Procedures*	$50 ($5)	$53 ($12)	-$4 (-$32 - $18)	0.776	$58 ($7)	$51 ($9)	$7 (-$16 - $27)	0.531
*Pathology and Laboratory Procedures*	$19 ($2)	$14 ($2)	$4 (-$2 - $10)	0.142	$18 ($2)	$15 ($2)	$3 (-$2 - $9)	0.248
*Radiology Procedures*	$14 ($2)	$14 ($3)	$1 (-$6 - $6)	0.833	$19 ($2)	$19 ($3)	-$1 (-$8 - $6)	0.898
*Surgery*	$71 ($10)	$53 ($7)	$18 (-$5 - $44)	0.142	$66 ($7)	$69 ($9)	-$3 (-$27 - $19)	0.798
*Other*	$21 ($2)	$24 ($4)	-$2 (-$12 - $5)	0.578	$72 ($36)	$40 ($14)	$32 (-$27 - $118)	0.398
Total Medicare Payments	$449 ($34)	$394 ($43)	$55 (-$56 - $161)	0.326	$808 ($246)	$801 ($238)	$8 (-$681 - $730)	0.985
Patient Copayment	$82 ($6)	$77 ($9)	$4 (-$17 - $25)	0.688	$109 ($15)	$99 ($11)	$10 (-$24 - $50)	0.624
Patient Deductible	$31 ($2)	$29 ($2)	$1 (-$4 - $7)	0.635	$29 ($2)	$38 ($10)	-$10 (-$33 - $5)	0.338
TPL Payments	$1 ($1)	$0 ($0)	$1 ($0 - $2)	0.193	$10 ($10)	$0 ($0)	$9 ($0 - $29)	0.203

CI = confidence interval; DME = durable medical equipment; ER = Emergency room; PPPM = per participant per month; SE = standard error; SNF = skilled nursing facility; TPL = third party liability

Notes: Payment data obtained from the Medicare claims data “Payment” cohort. Medicare payments were collected over the entire observation period (pre-study or on-study). The “Difference” column provides the A4 Medicare PPPM payment minus the LEARN Medicare PPPM payment within a category as well as the 95% CI of that difference.

⁎ Bold-faced numbers represent significant findings.

### 3.3 On-study period

#### 3.3.1 Clinical characteristics

During the on-study period, disorders of lipoprotein metabolism and other lipidemias and hypertension remained the most prevalent conditions in both cohorts, followed by malaise/fatigue, and sleep disorders (Table 1). A4 Medicare had a significantly higher proportion of participants with hypertension (A4: 73%, LEARN: 59%; OR 1.89 [95% CI 1.19-3.02]), memory loss (A4: 31%, LEARN: 18%; OR 2.02 [95% CI 1.17-3.49]), elevated blood glucose level (A4: 48%, LEARN: 37%; OR 1.58 [95% CI 1.00-2.49], and other disorders of the urinary system (A4: 38%, LEARN: 26%; OR 1.80 [95% CI 1.10-2.93]).

At the end of the on-study period, A4 Medicare had a significantly higher mean JFI score compared with LEARN Medicare (A4: 4.58, LEARN: 3.90; p=0.003) and a significantly different distribution according to level of frailty than LEARN Medicare, where a smaller proportion of A4 Medicare was categorized with low frailty (A4: 28%, LEARN: 44%; p=0.009) (Table 1). The JFI components associated with high frailty in A4 Medicare were minor ambulatory, dementia, self-care impairment, syncope, and cancer (**Suppl Table 4**).

#### 3.3.2 Cognitive assessments and diagnoses related to AD progression

By the end of the on-study period, the A4 and LEARN Medicare cohorts had significant differences in their unadjusted mean CDR-GS (A4: 0.22, LEARN: 0.06; p<0.001), CDR Sum of Boxes (CDR-SB) (A4: 0.79, LEARN: 0.13; p<0.001), Cognitive Function Index (CFI) (A4: 2.76, LEARN: 2.08; p=0.006), and MMSE scores (A4: 27.99, LEARN: 29.18; p<0.001) indicating greater cognitive decline in A4 Medicare (Table 1).

**Fig. 1:**
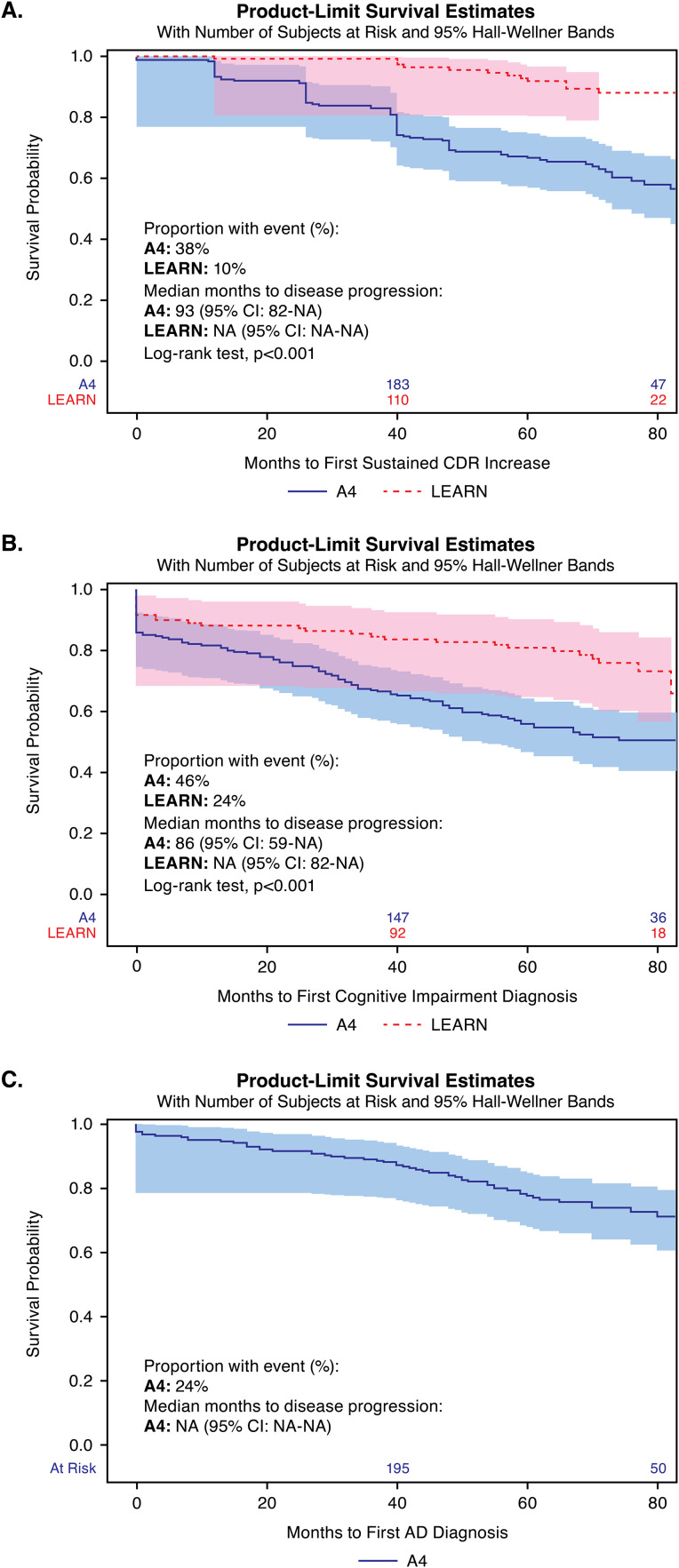
**Time from study entry to progression of preclinical Alzheimer’s disease in A4 and LEARN Medicare cohorts.** AD = Alzheimer’s disease; CDR-GS = Clinical Dementia Rating Scale-Global Score; CI = Confidence interval; CMS = Centers for Medicare & Medicaid Service; ICD = International Classification of Diseases; NA = Not available. Kaplan-Meier time from study entry to progression of preclinical AD in A4 and LEARN Medicare cohorts are shown in Panels A through C. Progression is defined as two consecutive, or one final, CDR-GS score(s) ≥0.5 in Panel A, as presence of an ICD-10 diagnosis code(s) for cognitive impairment in Medicare claims in Panel B, and as presence of an ICD-10 diagnosis code(s) for AD in Medicare claims in Panel C (median not reached). Note: Numbers above the x-axis are participants still at-risk for progression at those timepoints for each cohort. The proportion of participants with an AD diagnosis in LEARN Medicare was too low to include, per the CMS cell suppression policy.

Furthermore, each of the progression indicators were more prevalent in A4 vs LEARN Medicare: CDR-GS >0 (A4: 38% vs LEARN: 10%) or claims-based cognitive impairment diagnosis (A4: 46% vs LEARN: 24%), AD diagnosis (A4: 24% vs LEARN: suppressed low count), or high frailty (A4: 30% vs LEARN: 22%) (Fig. 1**A-C**).

Fig. 1**A-C** displays the unadjusted KM survival curves for time to first progression indicator during the on-study period. Many of the KM medians are not observed due to censoring. In A4 Medicare, 25% had CDR-GS progression by month 40 (Fig. 1**A**), 25% had a cognitive impairment diagnosis code(s) in claims by month 24 (Fig. 1**B**), and approximately 25% had AD diagnosis code(s) in claims by month 70 (Fig. 1**C**). In LEARN Medicare, approximately 25% received a cognitive impairment diagnosis by month 77 (Fig. 1**A**), but KM estimates for other progression indicators were not evaluable. Compared with LEARN Medicare, A4 Medicare participants were 4.25 times more likely to have CDR-GS progression (HR 4.5, 95% CI 2.32-7.79) and approximately 2 times more likely to have code(s) for cognitive impairment in claims (HR 2.13, 95% CI 1.40-3.24).

#### 3.3.3 HRU

A4 and LEARN Medicare had comparable inpatient, emergency room, and outpatient utilization in the on-study period (Table 2). The cohorts differed only in their annualized rate of encounters per 100 participants for non-emergency room outpatient visits, which was significantly lower in A4 vs LEARN Medicare (424 vs 545; p=0.002) and for pathology/laboratory procedures, which was significantly higher in A4 vs LEARN Medicare (277 vs 198, p=0.002) (Table 2).

#### 3.3.4 Medicare payment

Professional services represented the largest payment category for both A4 and LEARN Medicare in the on-study period. A4 and LEARN Medicare had comparable average PPPM total payments in the on-study period (A4 mean PPPM=$808; LEARN mean PPPM=$801; 95% CI difference in mean PPPM -$681-$730) (Table 3). No significant differences were observed within any payment category between A4 and LEARN Medicare (Table 3).

### 3.4 On-study associations between clinical outcomes, HRU, and Medicare payments and AD progression for A4 Medicare

#### 3.4.1 Clinical outcomes in A4 Medicare participants with disease progression

CDR-GS progression in A4 Medicare was associated with having a cognitive impairment ICD-10-CM diagnosis (OR=5.81, 95% 3.24-10.39) and with AD ICD-10-CM diagnosis (OR=4.64, 95% CI 2.41-8.93) in claims during the on-study period. These claims indicators of cognitive impairment or AD were associated with high frailty based on the JFI score ≥6 (cognitive impairment diagnosis OR: 2.64, 95% CI 1.48-4.71 and AD diagnosis OR: 2.13, 95% CI: 1.13-4.01). The end of the on-study period CDR-GS was positively correlated with each indicator of cognitive or functional decline in Medicare (**Suppl Table 5)**. For A4 Medicare participants whose CDR-GS indicated AD progression during the on-study period, 73% also had a cognitive impairment diagnosis in Medicare claims, 41% had an AD diagnosis, and 36% had high JFI frailty (**Suppl Table 6)**.

#### 3.4.2 HRU outcomes in A4 Medicare participants with disease progression

A4 Medicare participants with AD diagnosis code(s) in claims during the on-study period were more than twice as likely as those without an AD code to have any inpatient utilization (OR=2.24, 95% CI 1.20-4.17) (**Suppl Fig. 1**). A similar finding was observed for increased inpatient utilization among A4 Medicare participants with high JFI high frailty (scores ≥6) compared with those who had lower JFI frailty (scores <6) (OR=2.14, 95% CI 1.19-3.88). Inpatient emergency room use was higher among those with AD diagnosis code(s) in claims than those without code(s) (OR=2.50, 95% CI 1.21-5.17) and among those with JFI high frailty than those with lower JFI scores (OR=2.46, 95% CI 1.22-4.96), while outpatient emergency room use was higher among those with an AD diagnosis code(s) than those without AD code(s) (OR=2.24, 95% CI 1.17-4.28). Home health utilization was also higher for those with AD diagnosis code(s) than those without AD code(s) (OR=2.33, 95% CI 1.16-4.69) and those with JFI high frailty than those with lower JFI scores (OR=2.35, 95% CI 1.18-4.68). A4 Medicare participants with cognitive impairment diagnosis code(s) were almost twice as likely as those without cognitive impairment code(s) to have high (upper quartile rates of number of episodes) outpatient emergency room utilization (OR=1.99, 95% CI 1.08-3.64). A4 Medicare participants with AD diagnosis code(s) were more than twice as likely as those without an AD code to have high inpatient utilization (OR=2.59, 95% CI 1.35-5.00), as were those with JFI high frailty compared with those who had lower JFI scores (OR=2.74, 95% CI 1.46-5.14).

#### 3.4.3 Payment outcomes in A4 Medicare participants with disease progression

**Fig. 2:**
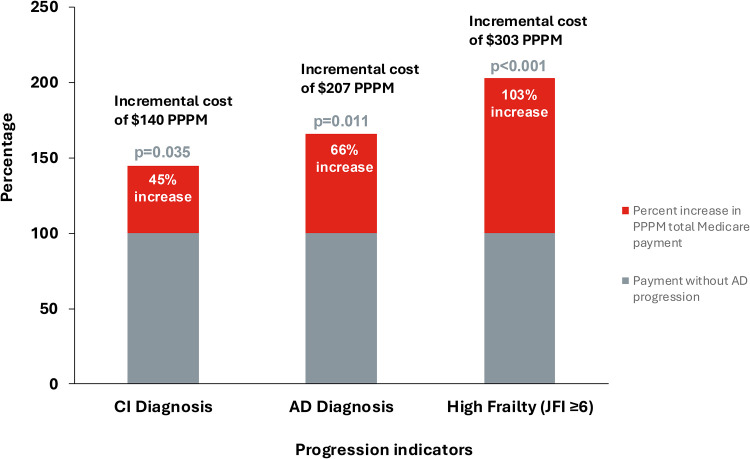
Increase in PPPM total Medicare payment associated with indicators of AD progression*. AD = Alzheimer’s disease; CI = cognitive impairment; FFS = fee for service; PPPM = per participant per month. *Restricted to individuals with Medicare FFS enrollment in the on-study period; percent increase estimated from the exponent of model estimates obtained from linear regression models predicting log-transformed PPPM total Medicare payments from each indicator after adjusting for age and sex; payments were inflation adjusted to 2021 dollars, and extreme outliers were Winsorized to the 99th percentile.

The PPPM total Medicare spending for A4 Medicare participants with cognitive impairment diagnosis code(s) during the on-study period was higher than those without cognitive impairment code(s) by a factor of 45%, after adjusting for age and sex: cost ratio 1.45 (95% CI 1.03–2.04), resulting in an incremental cost of $140 PPPM (95% CI $125-$155) (Fig. 2; **Suppl Table 7; Suppl Table 8**). For A4 Medicare participants with an AD diagnosis code(s) during the on-study period, a 66% increase in Medicare spending was observed compared with A4 Medicare participants without AD code(s): cost ratio 1.66, 95% CI 1.14–2.42, resulting in an incremental cost of $207 PPPM (95% CI $188-$226). A4 Medicare participants with high frailty (JFI ≥6) at the end of the on-study period were estimated to have a 103% increase in Medicare spending compared with those with lower JFI scores: cost ratio 2.03, 95% CI 1.41-2.92, resulting in an incremental cost of $303 PPPM (95% CI $283-$323). No statistically significant association of CDR-GS progression with PPPM total Medicare payments was observed in A4 Medicare.

Inpatient utilization and emergency room visits represented the largest payment increases in participants who had cognitive impairment, AD, and JFI high frailty in claims when compared with those who did not have cognitive impairment, AD, and JFI high frailty (**Suppl Table 7**). Outpatient services were another substantial contributor to higher payments (i.e., greater than 100% increase in Medicare payments) in those with JFI high frailty compared with those with lower JFI scores (**Suppl Table 7**).

## 4 Discussion

The A4 trial and LEARN study data linked with Medicare data provided a unique opportunity to study the impact of AD pathology and AD progression on HRU and Medicare spending in individuals with known amyloid status and no cognitive impairment at baseline. This is the first study, to our knowledge, of HRU and Medicare payments in cognitively unimpaired (preclinical) AD, with biomarker confirmation of AD pathology. The presence of AD pathology without cognitive impairment cognitively unimpaired AD) in A4 Medicare did not lead to increased HRU or payments during the on-study period, as similar HRU and payments were observed between A4 and LEARN Medicare. However, progression of cognitively unimpaired AD based on Medicare data (codes for cognitive impairment, AD, or high frailty) was associated with significantly and substantially higher HRU and payments in A4 Medicare. AD progression as measured by these claims indicators of cognitive or functional decline was often associated with higher inpatient, outpatient emergency room, and home health utilization (any utilization) as well as increased inpatient episodes (number of inpatient stays). Higher payments for these claims-based AD progression indicators were substantial, with mean incremental costs ranging from $140 PPPM to $303 PPPM, which translates to $1,680 to $3,636 per individual on an annual basis when compared with those who did not have these indicators.

Healthcare costs have been shown to additionally increase with further disease progression and greater level of impairment in later stages of AD [16,17]. In a study of Medicare claims in community-dwelling older adults, cognitive impairment (any etiology including AD and non-AD pathology) was associated with substantial incremental total and sector-specific healthcare expenditures [18]. Results from the Health and Retirement Study-Medicare population showed increased HRU (e.g., outpatient emergency department, skilled nursing, and home health) and total health care costs prior to clinical manifestation and diagnosis of AD [19]. A 36-month, U.S.-based, prospective, longitudinal cohort study (GERAS-US) found that total societal costs were higher for patients with AD with mild dementia compared with those with AD with MCI over 36 months (least squares mean total cost at baseline to 36 months: $4,063 to $3,392 for AD with mild dementia vs $2,430 to $2,007 for AD with MCI) [20]. Care partner indirect costs including costs based on informal care partner time and work loss were also higher for patients with AD with mild dementia compared with patients with AD with MCI (cost at baseline to 36 months: $1,953 to $1,964 for AD with mild dementia vs $718 to $961 for AD with MCI) [20]. A subgroup analysis of the GERAS-US study found that slowing progression of AD by 30% resulted in substantial reductions in care partner time and total societal costs (AD with MCI: $2,638; AD with mild dementia: $3,974) [21].

The increased costs associated with symptomatic stages of AD have been shown to largely be associated with related comorbidities. A retrospective cohort study (2014-2019) utilizing MarketScan Commercial and Medicare Supplemental Databases found that the economic burden of AD with MCI was two-folder higher than individuals without MCI or dementia due to greater emergency department, outpatient, and pharmacy costs [34]. Comorbidities were more common in individuals with AD with MCI vs the control cohort, which likely influenced cost. A similarly designed study reported that individuals who progressed from AD with MCI to AD and related dementia disorders had significantly higher health care costs, primarily due to inpatient visits and higher comorbidity burden, than individuals with stable MCI [35]. Another study similarly showed that AD is associated with increased Medicare claims/costs (e.g., inpatient and SNF services), with the primary diagnosis on the claim reflecting dementia-related comorbidities, such as vascular disease, infections, and injuries, rather than cognitive impairment [36]. These studies show the increased costs of comorbidities with each stage of AD, representing an opportunity for health care providers to diagnose and treat AD earlier to delay progression and reduce these associated costs.

It is notable that the claims indicators in this study but not the trial measurement of progression using CDR-GS were associated with statistically significant higher HRU and payments. This raises an important question: at what level of cognitive decline do HRU and costs increase? While the A4 trial indicator of AD progression measured using CDR-GS was not directly linked to higher HRU and payments in this study, it was associated with having cognitive impairment and/or AD code(s) in Medicare claims. The CDR-GS represents a standardized cognitive assessment administered in the context of the A4 and LEARN studies. There is some evidence that claims indicators may indicate a higher threshold of cognitive impairment than standardized cognitive assessments. For example, in a recent survey, physicians in the U.S., Japan, and several countries in Europe tended to rate patients with AD as less severe than their MMSE scores would indicate [37]. It should also be noted that CDR-GS progression is defined by increases to scores ≥0.5, which is the minimum score to detect cognitive decline. Thus, CDR-GS progression may provide an earlier indicator of progression before related diagnosis codes appear in the patients’ medical or claims record.

### 4.1 Strengths and limitations

This analysis provides unique insights into patterns of HRU and associated Medicare payments in the preclinical stage of AD, comparing a well characterized cohort of individuals with cognitively unimpaired AD (cognitively unimpaired with evidence of amyloid pathology in the A4 trial) with otherwise similar individuals without elevated amyloid (in LEARN). Linkage of the study and Medicare claims data sources allows for richer characterization of the earliest stages of AD including its economic outcomes.

Several limitations deserve mention when interpreting the findings of this study: 1) the number of participants was small—246 A4 participants and 121 participants in the LEARN study—limiting assessment of heterogeneity of the trajectory of decline and related costs; 2) the analysis was limited to the pre-study period and the on-study period but did not include data from the post-study period as these data continue to be accrued. Accordingly, the data presented do not provide reliable inferences about long-term cost; and 3) participants were predominantly White, thus limiting inferences to non-White individuals.

The Medicare cohorts for A4 and LEARN were demographically and clinically similar to the larger parent cohorts (e.g., family history of dementia, *APOE* ε4 status, and baseline cognitive and functional scores, and CDR-GS time to progression) (see [23] for A4, [11] for A4 and LEARN). However, these findings may be more generalizable to individuals with cognitively unimpaired AD likely to seek evaluation and treatment in clinical trials than to the broader cognitively unimpaired AD population. As Sperling noted, participants in such studies tend to be older, proactive about their health, and motivated by memory concerns or family history of AD [5]. Indeed, most participants in A4 and LEARN Medicare were ≥70 years old, 73% reported a family history of dementia, and more years of education (mean 17 years) than the general U.S. population aged ≥65 years (mean 12 years) [38]. Furthermore, it should be noted that some A4 study participants received an anti-amyloid investigational study drug (solanezumab) in the A4 trial even though the A4 trial found that solanezumab did not significantly slow cognitive decline compared with placebo over a period of 240 weeks, indicating it was not efficacious in this stage of the disease [23]. In addition, Vermunt and colleagues [39] found that cognitively unimpaired AD progressed more slowly in research observational study cohorts than clinical routine care settings. These observations may potentially limit generalizability of the A4 trial population to the overall population with cognitively unimpaired AD.

Because the protocol eligibility criteria excluded participants with a high level of medical comorbidity, our study cohorts represent a relatively healthier trial participants compared with a routine care population. Medical comorbidities, including those captured by the JFI such as cardiovascular conditions, syncope (e.g., increased likelihood of falls), and cancer, may have an impact on cognitive or functional decline and economic outcomes. Future similar studies could consider evaluating the impact of individual comorbidities or multimorbidity on disease progression and economic outcomes in larger real-world (routine care) cohorts of individuals with cognitively unimpaired AD and including components from the Charlson Comorbidity Index, multimorbidity-weighted index (MWI), JFI, or other comorbidity indices such as the Cumulative Illness Rating Scale-Geriatric [40,41]. Future studies could also evaluate disease progression and economic impact in commercial claims where utilization and payment profiles may be different in younger populations who have potentially fewer comorbidities.

The limitations of observational study designs apply here, including the potential for miscoding (particularly undercoding) of cognitive impairment and AD in Medicare claims [42]. Compliance with the CMS cell size suppression policy limited the ability to fully report all statistics.

### 4.2 Conclusion

Cognitively unimpaired AD participants in A4 Medicare showed increased clinical and cognitive decline and shorter time to progression compared to LEARN Medicare with over 5 years of follow-up (on-study period) but did not show increased HRU and payment. However, disease progression indicators observed in A4 Medicare were associated with higher HRU in multiple settings of care and higher Medicare payments. Future effective treatment that slows the disease progression of cognitively unimpaired AD may help individuals who have cognitively unimpaired AD to not only maintain cognition and functional independence but also enable them and the healthcare system to avoid some of the increased costs associated with decline. Future research should evaluate the longer term and broader health and economic impact of progression (e.g., indirect societal costs) ideally in a real-world population of individuals with cognitively unimpaired AD and the impact of future effective disease-modifying therapies for avoiding increased costs observed with progression of cognitively unimpaired AD.

No Generative AI and AI-assisted technologies was used in the writing process.

## Funding

This work was supported by Eli Lilly and Company, Indianapolis, IN, USA

## Role of the Funder/Sponsor

The sponsors provided guidance for the conduct of the research and the preparation of the article; the collection, analysis, and interpretation of data; the writing of the report; and the decision to submit the article for publication.

## Data Sharing

Per the informed consent signed by study participants, the Medicare data for these study cohorts cannot be shared publicly. All results presented rely on fully compliant analyses conducted within the terms of the informed consent and CMS policy.

## CRediT authorship contribution statement

**Julie Beyrer:** Writing – review & editing, Writing – original draft, Supervision, Project administration, Investigation, Conceptualization. **Zachary Sheff:** Writing – review & editing, Writing – original draft, Supervision, Investigation, Conceptualization. **Nalin Payakachat:** Writing – review & editing, Writing – original draft, Supervision, Project administration, Investigation, Conceptualization. **Julie M. Chandler:** Writing – review & editing, Investigation, Conceptualization. **Yun-Fei Chen:** Writing – review & editing, Formal analysis. **Joanna Kubisiak:** Writing – review & editing, Formal analysis. **Angelina Lee:** Writing – review & editing, Formal analysis. **Karen C. Holdridge:** Writing – review & editing. **Roy Yaari:** Writing – review & editing, Investigation. **Paul Aisen:** Writing – review & editing, Investigation. **Michael S. Rafii:** Writing – review & editing, Investigation. **Reisa A. Sperling:** Writing – review & editing, Investigation.

## Declaration of competing interest

The authors declare the following financial interests/personal relationships which may be considered as potential competing interests:

Julie Beyrer reports financial support was provided by Eli Lilly and Company. Julie Beyrer, Zachary Sheff, Nalin Payakachat, Julie, M. Chandler, Yun-Fei Chen, Karen C. Holdridge, and Roy Yaari reports a relationship with Eli Lilly and Company that includes: employment and equity or stocks. Joanna Kubisiak and Angelina Lee reports a relationship with Westat Inc that includes: employment. Paul Aisen, Michael S. Rafii, and Reisa A. Sperling reports a relationship with Eli Lilly and Company that includes: funding grants. If there are other authors, they declare that they have no known competing financial interests or personal relationships that could have appeared to influence the work reported in this paper.

## Appendix Supplementary materials

Image, application 1
