# Supplementary material for: Increase in healthcare utilization and Medicare payment with progression of preclinical Alzheimer’s disease
Source: J Prev Alzheimers Dis. 2026 Apr 1;13(6):100547. doi: 10.1016/j.tjpad.2026.100547 (PMC13084671; doi:10.1016/j.tjpad.2026.100547)

**Supplementary Data**

| **Suppl Table 1. Data reported for the analytic cohorts** | | | | | | |
| --- | --- | --- | --- | --- | --- | --- |
| **Addendum Cohort** | **Type of Medicare Enrollment(s) Required*** | **Reported Information for Cohorts** | | | | |
|  |  | **A4 and LEARN Data** | **Medicare Data** | | | |
|  |  | **Demographic Information and Assessments** | **Enrollment and Demographic Information** | **Chronic Conditions and Diagnoses** | **Utilization** | **Payment** |
| Utilization Cohort | Medicare Parts A&B with FFS coverage or MA plan participation after 2014^†^ | X | X | X | X |  |
| Payment Cohort | Medicare Parts A&B with FFS coverage |  |  |  |  | X |
| FFS = Fee-for-service, MA = Medicare Advantage  *At least one month of qualifying Medicare enrollment(s) is required for inclusion; analyses include FFS data through 2022 and MA data through 2021.  ^†^MA data is available starting in 2015. | | | | | | |

| **Suppl Table 2.** **ICD-9 and ICD-10 diagnosis codes for identifying cognitive impairment and AD in the Medicare data*** | |
| --- | --- |
| ICD-9 Diagnosis Codes for Cognitive Impairment | 2900, 29010, 29011, 29013, 29021, 2903, 29040, 29041, 29042, 29043, 29410, 29411, 29420, 29421, 2949, 31081, 31089, 33111, 33119, 3312, 3317, 33182, 33183, 33189, 3319, 34830, 4380, 78009, 78093, 797 |
| ICD-9 Diagnosis Codes for AD | 3310 |
| ICD-10 Diagnosis Codes for Cognitive Impairment | F0150, F0151, F0280, F0281, F0390, F0391, F068, F0789, F482, G132, G138, G3101, G3109, G311, G312, G3181, G3182, G3183, G3184, G3185, G3189, G319, G914, G9340, G94, I69010, I69011, I69014, I69015, I69018, I69110, I69111, I69114, I69115, I69118, I69210, I69211, I69214, I69215, I69218, I69310, I69311, I69314, I69315, I69318, I69810, I69811, I69814, I69815, I69818, I69910, I69911, I69914, I69915, I69918, R400, R401, R412, R413, R4181, R54 |
| ICD-10 Diagnosis Codes for AD | G300, G301, G308, G309 |
| AD = Alzheimer’s disease; CMS = Centers for Medicare & Medicaid Services; GEM = General Equivalence Mappings; ICD = International Classification of Diseases  *The list of diagnosis codes used to identify cognitive impairment/AD in the Medicare data was informed by a previous study conducted by JEN Associates, Inc. (now Westat) and Eli Lilly on the diagnostic pathways to AD, and updated after review by Eli Lilly clinical consultants. The original list consisted of ICD-9 diagnoses. These codes were mapped to ICD-10 codes using CMS GEMs. | |

| **Suppl Table 3. Demographic and other baseline characteristics from Medicare enrollment data of the A4 and LEARN Medicare cohorts** | | | |
| --- | --- | --- | --- |
| **Baseline Characteristic** | **Percent or  Mean (SE)** | **Percent or  Mean (SE)** | **p-value** |
|  | **A4 trial**  **(N=246)** | **LEARN study**  **(N=121)** |  |
| **Sex** |  |  |  |
| Female | 56% (3%) | 60% (5%) | 0.508 |
| Male | 44% (3%) | 40% (5%) |  |
| **Age (in year of study entry)** |  |  |  |
| Age in year of study entry (mean) | 72.3 (0.3) | 70.8 (0.4) | **0.004*** |
| Age in year of study entry: 65 to 69 | 33% (3%) | 45% (5%) | **0.022*** |
| Age in year of study entry: 70 to 74 | 34% (3%) | 36% (4%) |  |
| Age in year of study entry: 75 plus | 33% (3%) | 19% (4%) |  |
| **Race** |  |  |  |
| White | 95% | 91% | 0.081 |
| Non-white | 5% | 9% |  |
| **Education** |  |  |  |
| Means years of education | 16.7 (0.2) | 17.4 (0.2) | **0.009*** |
| **Marital status** |  |  |  |
| Married | 73% (3%) | 65% (4%) | 0.119 |
| Divorced or other | 27% (3%) | 35% (4%) |  |
| **Retirement status** |  |  |  |
| Retired | 80% (3%) | 75% (4%) | 0.245 |
| Not retired | 20% (3%) | 25% (4%) |  |
| **Region** |  |  |  |
| Northeast | 30% (3%) | 49% (5%) | **<0.001*** |
| Midwest | 31% (3%) | - |  |
| South | 30% (3%) | - |  |
| West | 10% (2%) | - |  |
| **NCHS Urban-Rural County Classification** |  |  |  |
| Large Central Metro | 27% (3%) | 37% (5%) | **0.011*** |
| Large Fringe Metro | 36% (3%) | 39% (5%) |  |
| Medium Metro | 18% (3%) | - |  |
| Nonmetro-Small Metro | 19% (3%) | - |  |
| **Family History of Dementia—parent or sibling** |  |  |  |
| Family History—parent or sibling with dementia or significant memory impairment | 76% (3%) | 66% (4%) | 0.045 |
| ***ApoE* ε4 carrier status** |  |  |  |
| *ApoE* ε4 carrier | 62% (3%) | 12% (3%) | <0.001 |
| NCHS = National Center for Health Statistics; SE = standard error  Notes: Demographic data obtained from Medicare claims data “Utilization” cohort (education data obtained from A4/LEARN study data) and collected at study entry in the on-study period. No standard errors were reported for data on race. Cells containing less than 11 individuals are suppressed per Medicare policy and denoted with a dash.  *Bold-faced numbers represent significant findings. | | | |

| **Suppl Table 4. Components of the JEN Frailty Index (JFI) associated with having a High End of Study JFI Score (6+) among A4 Medicare Study Participants in the on-study Utilization Cohort** | | | | |
| --- | --- | --- | --- | --- |
| **JFI Impairment/ Frailty Category** | **Chi-square p-value** | **Odds Ratio and 95% CI** | | |
|  |  | **Odds Ratio**^†^ | **Lower confidence limit** | **Upper confidence limit** |
| Minor Ambulatory | **0.037*** | 1.91 | 1.04 | 3.51 |
| Severe Ambulatory | 0.450 | 1.80 | 0.39 | 8.25 |
| Mental Illness | 0.862 | 1.09 | 0.40 | 3.00 |
| Dementia | **0.037*** | 2.89 | 1.07 | 7.82 |
| Sensory Limitations | 0.056 | 4.14 | 0.96 | 17.83 |
| Self-care Impairment | **0.004*** | 3.48 | 1.49 | 8.12 |
| Syncope | **0.034*** | 2.32 | 1.06 | 5.05 |
| Cancer | **0.025*** | 5.05 | 1.23 | 20.78 |
| Chronic Medical Disease | 0.248 | 1.62 | 0.72 | 3.66 |
| Renal Disorders | 0.069 | 4.93 | 0.88 | 27.53 |
| Other Systemic Disorders | 0.293 | 1.72 | 0.63 | 4.71 |
| Developmental Disability | N/A |  |  |  |
| Pneumonia | N/A |  |  |  |
| N/A = not available  *Bold-faced numbers represent significant findings.  ^†^Each of the JFI components was analyzed separately using logistic regression for the binary outcome (JFI score of ≥6 versus <6) with adjustments for age and sex during the participant’s final on-study month and previous 11 months. | | | | |

| **Suppl Table 5. Comparison of end of on-study CDR Global scores between A4 Medicare participants in the on-study utilization cohort with and without indicators of AD progression** | | | | | | | |
| --- | --- | --- | --- | --- | --- | --- | --- |
|  | **End of On-study CDR Global Scores** | | | | **Spearman Correlation** | | |
|  | **Progression Indicator = No** | | **Progression Indicator = Yes** | | |  |  |
| **AD Progression Indicator** | **Mean** | **SE** | **Mean** | **SE** | | **Coefficient** | **p-value** |
| Cognitive Impairment Diagnosis | 0.1047 | 0.0180 | 0.3486 | 0.0366 | | 0.3857 | **0.000*** |
| AD Diagnosis | 0.1492 | 0.0171 | 0.4298 | 0.0606 | | 0.3211 | **0.000*** |
| End of Study High JFI Frailty | 0.1766 | 0.0200 | 0.3099 | 0.0505 | | 0.1447 | **0.026*** |
| AD = Alzheimer’s disease; JFI = JEN Frailty Index  *Bold-faced numbers represent significant findings. | | | | | | | |

| **Suppl Table 6. Proportion of A4 Medicare participants with AD progression identified by CDR Global scores and each claims indicator during the on-study period** | | | |
| --- | --- | --- | --- |
|  | **CDR-GS Progression Indicator** | | **Chi-square p-value** |
|  | **Progressor** | **Non-Progressor** |  |
| Cognitive Impairment Diagnosis | 72.5% | 29.3% | **0.000*** |
| AD Diagnosis | 40.7% | 13.6% | **0.000*** |
| End of Study High JFI Frailty | 36.3% | 25.9% | **0.088*** |
| AD = Alzheimer’s disease; CDR-GS = Clinical Dementia Rating Scale-Global Score; JFI = JEN Frailty Index  *Bold-faced numbers represent significant findings. | | | |

| **Suppl Table 7: Increase in PPPM Medicare payments associated with indicators of AD progression*** | | | | | | |
| --- | --- | --- | --- | --- | --- | --- |
|  | **CI Diagnosis** | | **AD Diagnosis** | | **High Frailty** | |
| **Payment Category** | **Percent Increase†** | **p-value** | **Percent Increase†** | **p-value** | **Percent Increase†** | **p-value** |
| Inpatient | 112% | 0.089 | 233% | **0.015**^‡^ | 149% | 0.063 |
| ER | 53% | **0.039**^‡^ | 81% | **<0.01**^‡^ | 132% | **<0.001**^‡^ |
| Outpatient | 42% | 0.223 | 35% | 0.352 | 116% | **0.014**^‡^ |
| DME | 23% | 0.363 | 0% | 0.989 | 90% | **0.009**^‡^ |
| Home Health | 1% | 0.973 | 71% | 0.075 | 38% | 0.282 |
| Professional Services | 33% | 0.060 | 44% | **0.033**^‡^ | 69% | **0.002**^‡^ |
| Total Medicare Payments | 45% | **0.035**^‡^ | 66% | **0.011**^‡^ | 103% | **<0.001**^‡^ |
| AD = Alzheimer’s disease; CI = cognitive impairment; DME = durable medical equipment; ER = emergency room; FFS = fee for service; PPPM = per participant per month  *Restricted to individuals with Medicare FFS enrollment in the on-study period.  †Percent increase estimated from the exponent of model estimates obtained from linear regression models predicting log-transformed PPPM payments from each indicator after adjusting for age and sex; payments were inflation adjusted to 2021 dollars, and extreme outliers were Winsorized to the 99th percentile.  ^‡^Bold-faced numbers represent significant findings. | | | | | | |

| **Suppl Table 8. Mean predicted PPPM payments by progression status*^,^**^†^ | | | | | |
| --- | --- | --- | --- | --- | --- |
| **Progression Measure** | **% Progressing in A4** | **Progression indicator = No** | **Progression indicator = Yes** | **Cost ratio** | **Mean Incremental Cost**  **(Progressors minus Non-progressors)^§^** |
|  |  | **Mean (95% CI)** | **Mean (95% CI)** | **Mean (95% CI)** | **Mean (95% CI)** |
| CDR-GS >0 | 38% | $348  (280, 431) | $364  (280, 472) | 1.05  (0.75, 1.46) | $16  (2, 30) |
| Cognitive impairment diagnosis | 46% | $310  (253, 380) | $450  (342, 591) | **1.45**  **(1.03, 2.04)**^‡^ | **$140**  **(125, 155)**^‡^ |
| AD diagnosis | 24% | $314  (261, 378) | $521  (374, 726) | **1.66**  **(1.14, 2.42)**^‡^ | **$207**  **(188, 226)**^‡^ |
| High frailty (JFI >6) | 30% | $296  (246, 356) | $599  (437, 821) | **2.03**  **(1.41, 2.92)**^‡^ | **$303**  **(283, 323)**^‡^ |
| AD = Alzheimer’s disease; CDR = Clinical Dementia Rating Scale-Global Score; JFI = JEN Frailty Index; PPPM = per participant per month  *For the linear payment models, two modeling approaches were considered: (1) log-normal model, using log-transformed dependent payment variables assuming a normal distribution, as outlined in the final report, and (2) log-link model, using untransformed payment variables assuming a gamma distribution with a log-link function, following the methodology from Sheets, et al., 2025. The residuals from the log-normal model appeared more symmetrically distributed and closer to normality, with less heteroscedasticity than the log-link model. Additionally, the Akaike Information Criterion was lower for the log-normal model, indicating a better overall model fit. These diagnostics suggest that the log-normal model is a more appropriate model choice for the data. To be consistent with the payment analyses in the final report, PPPM payments is the unit analyzed; payments do not include the patient and third party liability amounts. Additionally, special Medicare payments were not included (inpatient pass-through payments, Indirect Medical Education amounts, and Disproportionate Share amounts); not all facilities receive these payments, and they can obscure the true cost of healthcare services. For example, CMS excludes these payments from analyses of the Medicare Shared Saving Programs.  ^†^Models include all participants, regardless of whether they have payments; cells in bold correspond to Figure 10 (Percent Increase in PPPM Total Medicare Payment associated with Indicators of AD Progression) in the final report.  **^§^**Confidence intervals were estimated using the pooled standard error based on the following equations:  sp = sqrt{ [ (n_1_ -1) * s_1_^2^) + (n2 -1) * s_2_^2^) ] / (n_1_ + n_2_ - 2) }  SE = sp * sqrt( 1 / n_1_ + 1 / n_2_ )  where s_p_ is the pooled estimate of the equal population standard deviations, s_1_ and s_2_ are sample standard deviations, and n_1_ and n_2_ are sample sizes.  The Welch-Satterthwaite Approximation was used to find degrees of freedom for the t-score:  num = (s_1_^2^/n_1_ + s_2_^2^/n_2_)^2^  den = [(s_1_^2^/n1)2/(n_1_ - 1)] + [(s_2_^2^/n_2_)^2^/(n2 - 1)]  df = num/den  where s_1_^2^ and s_2_^2^ are the sample variances for the two groups, and n_1_ and n_2_ are the sample sizes for the two groups. (If this formula produces a non-integer df, round df down to the nearest whole number.)  ^‡^Bold faced represents significant findings. | | | | | |

**Suppl Figure 1. Association between indicators of AD progression and types of Medicare utilization**

**Note:** The relationship between HRU and the varying indicators of AD progression is shown in the figure. These analyses focused on the setting most utilized by participants, where any utilization is defined by the number of participants with any utilization in that setting, and where high utilization is defined by those with annualized PPPM rates in the upper quartile of the A4 Medicare cohort (bottom 2 figures). Odds ratios and 95% confidence intervals summarized the likelihood of having different types of utilization in relation to the different indicators of cognitive or functional decline.


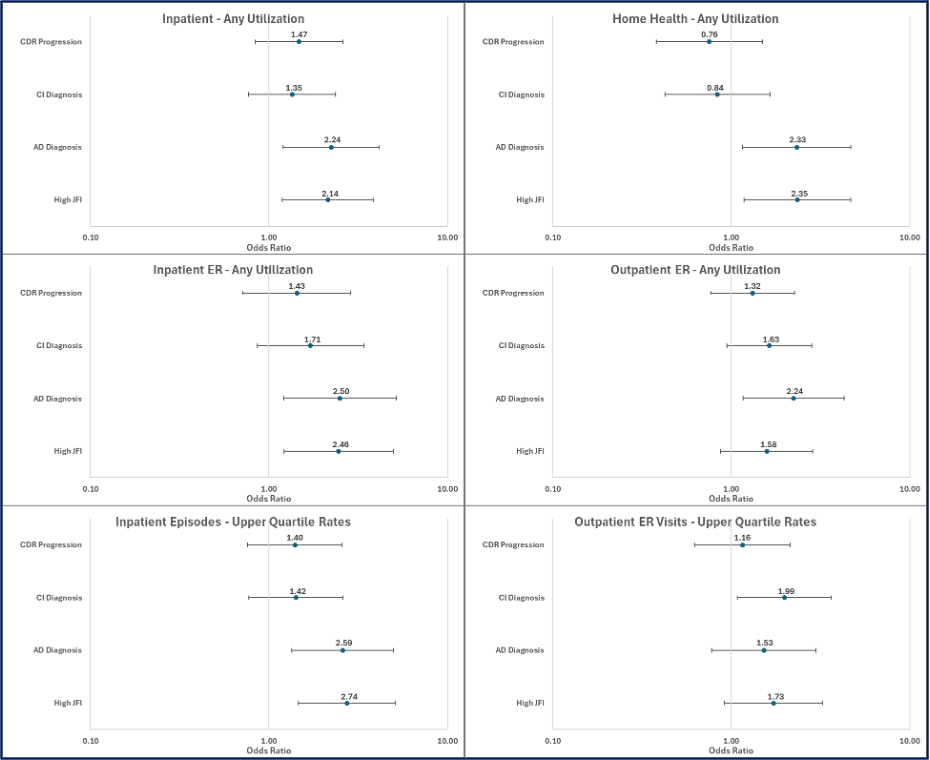

Supplement: Supplementary file 1 [file mmc1.docx]
